# Climate Change and Youth: Fast-Tracking Mental Health Solutions

**DOI:** 10.1007/s43151-025-00174-1

**Published:** 2025-04-07

**Authors:** Caroline X. Gao, Nicholas Fava, Vivienne Browne, Rebecca Patrick, Hasini Gunasiri, Jana M. Menssink

**Affiliations:** 1 Centre for Youth Mental Health, The University of Melbourne, Parkville, VIC Australia; 2 Orygen, Parkville, VIC Australia; 3 Department of Epidemiology and Preventative Medicine, School of Public Health and Preventive Medicine, Monash University, Melbourne, VIC Australia; 4 Melbourne School of Population and Global Health, The University of Melbourne, Parkville, VIC Australia

**Keywords:** Climate change, Mental health, Youth, Extreme weather events, Interventions, Policymaking

## Abstract

Worldwide, we are facing escalating mental health impacts related to climate change. Young people are disproportionately affected by climate-related extreme weather events such as severe heatwaves and floods. As climate change accelerates, increased frequency and severity of these weather events, along with awareness of the global environmental crisis, contribute to rising levels of hopelessness, anxiety, and distress among young people. This emotional burden is compounded by young people’s exclusion from decision-making processes. Interventions targeting the mental health impacts of climate change are still in their infancy. We call for innovative research designs, such as adaptive platform trials, to rapidly evaluate and implement youth-specific mental health interventions in the context of climate change. We highlight the importance of a whole-of-society approach, involving collaboration between young people, researchers, clinicians, educators, and communities to develop tailored interventions. Governments and research bodies should prioritise dedicated funding for climate change and mental health research at local, national, and global levels. Moreover, it is essential to integrate youth perspectives into policymaking and strengthen climate action to mitigate mental health impacts.

## Commentary

Climate change is accelerating, evidenced by recent record-breaking temperatures, unprecedented heat, droughts, bushfires, and floods across the globe (IPCC 2023). The resulting health impacts are intensifying worldwide, affecting billions of people (Romanello et al. 2023). Climate change not only negatively affects physical health, but a growing body of research also suggests that it is worsening people’s mental health and emotional well-being (Burrows et al. 2024; Charlson et al. 2021; Cianconi et al. 2020). Climate-related extreme weather events and disasters (e.g., heatwaves, storms, bushfires, floods, and droughts) as well as awareness of the threat of climate change can lead to a range of negative emotions, such as anxiety, distress, worry about the future, trauma, feelings of hopelessness, powerlessness, ecological grief, and anger stemming from injustice and perceived inaction from governments, industry, and other key stakeholders (Clayton 2020; Crandon et al. 2022; Gunasiri and Haddock 2023; Gunasiri, Wang, et al., 2022; Hickman et al. 2021; Patrick et al. 2022). Other indirect effects of climate change, such as physical ill-health, housing instability and displacement, water and food insecurity, social fragmentation, and conflicts, are all stressors for poor mental health (Berry et al. 2010).

Young people bear the greatest psychological burden associated with the impacts of climate change (see reviews by Koder et al. 2023; Patrick et al. 2022; White et al. 2023). According to an international survey of youth aged 16 to 25 (*N* = 10,000), almost half described how they felt about climate change impacting their functioning on a daily basis, and three-quarters felt scared about the future (Hickman et al. 2021). This disproportionate impact is partly due to young people’s critical developmental stage, where heightened emotional sensitivity and identity formation can be easily disrupted by climate change and related events, leading to stress, anxiety, trauma, uncertainty, and a pessimistic outlook on the future (McGorry et al. 2025; Teo et al. 2024). This is compounded by their exclusion from policymaking and global actions, leaving them feeling powerless despite being advocates for change. Research has linked climate anxiety to perceived government inaction, with young people experiencing frustration, mistrust, and betrayal due to tokenistic participation, criticism of youth activism, and political and corporate inaction (Bowman and Pickard 2021; Hickman et al. 2021; Pickard 2022). Systemic and intergenerational inequities further exacerbate this burden, as marginalised youth face compounding stressors like displacement and financial difficulties, while inheriting the consequences of insufficient action by previous generations, deepening their sense of injustice and hopelessness (White et al. 2023; Hickman et al. 2021).

While these emotional responses are in line with expected sociocultural norms in the context of climate change being a global threat, a rapidly growing body of evidence highlights that these emotions play critical roles in affecting young people’s mental health and well-being (Boluda-Verdú et al. 2022; Clayton 2020; Crandon et al. 2022; Fava et al. 2023a, 2023b; Gago et al. 2024; Gunasiri and Haddock 2023; Gunasiri et al. 2022a, b; Gunasiri, Wang, et al., 2022; Sciberras and Fernando 2022; Teo et al. 2024). These negative emotions are also increasingly compounded by exposure to more severe and frequent climate-related disasters resulting in more intense and complex mental health outcomes (Climate Council 2023; Gao et al. 2024; Gunasiri et al. 2022a, b; Hrabok et al. 2020; O’Donohue et al. 2022). Since the COVID-19 pandemic, there has been a rapid decline in the mental health of young people worldwide (Botha et al. 2023; Thiagarajan and Newson 2023). While various social and environmental factors have contributed to the current mental health crisis, climate change also plays an important role (Kirkbride et al. 2024; McGorry et al. 2025). Given the profound impacts of climate change on mental health among young people, urgent action is needed to address these challenges and build psychological resilience.

## The Challenges and Opportunities

There is a pressing need to understand and mitigate the direct and indirect impacts of climate change on youth mental health, not only for improving individual health outcomes but also for ensuring the resilience of communities and the broader health system in the face of ongoing environmental challenges (Seth et al. 2023). While there is increased awareness of the mental health issues related to climate change among young people, effective mitigation strategies are lacking (Ramadan et al. 2023). Research focused on interventions is still in its infancy. A recent literature review suggested the vast majority of available interventions were published in grey literature, with very little youth focus and no evidence of efficacy and effectiveness (Xue et al. 2024). Strikingly, across multiple clinical trial registries globally,^1^ only four interventions targeted at youth have been identified. Although many novel intervention approaches are emerging (Xue et al. 2024), the efficiency of knowledge translation within the existing research system is very low. It is estimated that only 14% of research findings are translated into care (Grimshaw et al. 2012; Morris et al. 2011), with an average lag of 17 years (Kessler & Glasgow 2011). This inefficiency puts young people at increasing risk of escalating mental health issues related to climate change.

The discrepancy between the urgent needs and the slow process of research translation into care highlights the necessity for innovative study design to fast-track the development and evaluation of interventions (Ryan et al. 2024). For instance, a stepped wedge cluster randomised trial design, which sequentially rolls out interventions to different clusters (e.g., schools) across time, can be employed to simultaneously implement the intervention and establish evidence of effectiveness (Hemming et al. 2015). The traditional two-arm trial design, which can only evaluate the effectiveness of a single intervention, can be replaced with more advanced designs that allow multiple interventions to be tested simultaneously within a single overarching trial structure. The most flexible of these designs is an adaptive platform trial, which allows interventions to continually enter or leave the platform, leading to faster and more robust conclusions about the effectiveness of multiple interventions (Angus et al. 2019; Berry et al. 2015). Such innovative designs have transformed evidence translation in physical health fields, such as COVID-19 treatment (Angus et al. 2020), and hold significant potential for accelerating the development of interventions for the mental health impacts of climate change.

Another challenge in developing interventions for the mental health impact of climate change is that there is no one-size-fits-all approach. Young people may present with different levels and types of impacts, ranging from mild climate distress to trauma- and stressor-related disorders secondary to climate disasters; therefore, the level of needs may differ depending on the characteristics and experiences of young people, intervention settings (e.g., schools, healthcare providers), and geographical location (Corvalan et al. 2022; Fava et al. 2023a). This requires a whole-of-society approach to promote innovation and collaboration between researchers, education sectors, environmental organisations, local communities, charity organisations, clinical services as well as other key stakeholders (Fava et al. 2023a). These initiatives may range from incorporating resilience and coping skills workshops into education on climate change in schools (Newberry Le Vay et al. 2023) to green social prescribing (Baudon and Jachens 2021).

The current mental health services and traditional therapeutic approaches do not consider the unique triggers and stressors brought about by climate change and related societal contexts (Seth et al. 2023; Xue et al. 2024). For example, although disaster psychiatry offers established interventions for post-traumatic climate disaster exposure, existing interventions often do not take climate change into account (Xue et al. 2024). Unfortunately, locations that have been impacted by climate-related disasters, such as bushfires or flooding, are often at increased risk of repeated events. Thus, individuals may experience repeated stressors, such as environmental destruction, loss of property, and deaths in their community, and greater mental and physical health impacts (Carroll et al. 2022). This highlights the need for better tailored clinical approaches to support those multiply at risk.

Another outstanding issue with the current limited interventions in climate mental health is the absence of a theoretical framework and evidence to support the mechanisms of change (Xue et al. 2024). There is increasing knowledge regarding potential mechanisms for effective interventions, such as validating emotional experiences, supporting social connection, increasing adaptive coping skills, and actively involving the community (Flies et al. 2023; Mah et al. 2020; Patrick et al. 2022; Patrick et al. 2023; Pitt et al. 2024). Emerging evidence suggests these approaches can reduce climate change-related negative emotions, support emotional and functional recovery from climate disaster impacts, and increase resilience against future disaster exposure (Block et al. 2019; Gibbs et al. 2021; Longman et al. 2023; Marinkovic Chavez et al. 2023). However, more research and theoretical development are needed. When designing and evaluating interventions, it is important for researchers to establish and evaluate potential key mechanisms for effectiveness. Importantly, negative emotions regarding climate change, such as worry and climate distress, are valid responses to a real threat (Bhullar et al. 2022). Responses to the mental health impact of climate change should not aim to reduce climate concern (Koder et al. 2023), but instead aim to support youth experiencing adverse impacts on wellbeing and functioning.

To effectively design and evaluate psychosocial interventions, there is also a critical need to develop appropriate and reliable measurement scales for both outcomes and mechanisms of change. Although diverse instruments measuring the mental health impact of climate change have been created, the validity of these tools remains poorly understood (Martin et al. 2023), with many carrying a pathological lens (e.g., Climate Anxiety scales) (Massazza et al. 2022) that tend to overlook those with mild-to-moderate impacts (Martin et al. 2023). Additionally, there is a lack of evaluation in the climate sector of positive mental health states (Massazza et al. 2022). Although various resilience and wellbeing measures exist (Kern et al. 2016; Liebenberg et al. 2011), they fail to encapsulate crucial climate-related aspects (e.g., perseverance, optimism, and connectedness), which are vital for targeted interventions (Massazza et al. 2022).

An important consideration in shaping the future landscape of psychosocial interventions in the context of climate change is the need to engage with the broader community and consumers, particularly those with Lived experience (of emotional impacts due to climate change and/or climate-related disasters), Loved experience (through the role as a caregiver), Laboured experience (of working in a mental health support role), and Learned experience (experience as a researcher), (4L, Killackey 2023). It is important to ensure that youth experiencing intersectional oppression, who are more likely to experience disproportionate impacts of climate change, are included in initiatives (Bessant et al. 2023; Vogel et al. 2022). A recent major global effort is the Connecting Climate Minds project, which has established a global research and action agenda based on the experiences and expertise of people with lived experience of mental health issues and of communities most affected by climate change across the world (Lawrance et al. 2024). However, more efforts are needed to establish 4L experience networks (both locally and globally) that can support the co-development of the most appropriate, fit-for-purpose, easy-to-implement interventions.

Working with young people in the context of climate change involves more than just consultation; it is about actively engaging them in decision-making processes, educational programs, and co-designing initiatives. Through these channels, young people can directly influence climate policies and mental health initiatives, ensuring that their voices are heard and their ideas integrated into decision-making. This engagement fosters a sense of agency, self-efficacy, and wellbeing, which are essential for their mental wellbeing (Berry 2021; Crowley and Moxon 2017; Hohenhaus et al. 2023). Peer-led advocacy and support systems play a crucial role, allowing young people to lead campaigns, raise awareness, and support their peers (Mark and Lewis 2020; Psychology for a Safe Climate 2023). Safe spaces, both physical and online, are necessary to allow young people to express their concerns, share experiences, and access mental health support, which can reduce isolation and build a sense of community (e.g., Museums Victoria 2024).

In these spaces, young people are framed as active contributors with innovative ideas, valuable perspectives, and the potential to drive change in both climate action and mental health. They are recognised as a priority population for climate adaptation policies due to their heightened vulnerabilities and are seen as leaders in building resilience. Their involvement in decision-making processes not only contributes to shaping a sustainable future but also provides them with a sense of purpose and belonging, which is crucial for their mental health and overall wellbeing.

## Policy Implications

Solutions are available to address the mental health impacts of climate change, requiring promptness to reflect its increasing threat and disproportionate impact on young people’s mental health. Given the need for interventions, the limited evidence-based interventions available and the extended time required to translate research to practice, there is a need for research funding bodies to develop dedicated funding calls for climate change and mental health projects. Funding rounds should:

- Focus on young people as a priority population;
- Promote research innovations and support translation, such as adaptive platform trials, co-designed interventions with lived experience stakeholders, identify key mechanisms for effectiveness of interventions, and develop reliable measurement scales; as well as
- Promote collaboration between researchers, clinical services, educational settings and community organisations.

While the evidence base for psychosocial interventions continues to grow, there are several key activities at a policy level that the government should consider. Young people have identified that government climate inaction is a key driver impacting their mental health (Hickman et al. 2021) and that seeing the government take strong climate action would be the most helpful solution for young people’s climate distress (Fava et al. 2023b).

Young people, a priority population in the context of climate change, have the right to be actively involved in decision-making processes at both local and international levels. Establishing youth advisory groups and integrating co-design elements in climate-related programs can foster meaningful climate action and address climate-related mental health issues by ensuring that young people’s perspectives and voices are central to the development and implementation of policies.

To better support research and practice innovations addressing the impact of climate change on young people, a supportive national and global policy environment is essential. For example, developing integrated health and climate strategies requires policymakers to take actions based on the best available evidence (CDC/ATSDR, 2022; Department of Health and Aged Care 2023). Governments are also responsible for monitoring and reporting progress towards emissions targets, climate change policy and their impact (e.g., the Annual Climate Change Statement in Australia). There is a valuable opportunity to enhance these reports by including information on the impacts of climate change on young people and their mental health. This could be informed by health impact assessments on all future climate and energy-related policies that assess the impact that potential policies and projects have on the health and wellbeing of young people and future generations. Additionally, an adapted Youth Annual Climate Change Statement and dissemination plan could be developed to ensure that young people are informed about government action on climate change. These actions ensure that young people’s health and wellbeing are considered across all climate-related policy and that young people remain regularly informed about government action on climate change.

## Conclusion

The health and economic burden related to climate-related extreme events is predicted to increase significantly over the next decade (IPCC 2023). While awareness of health impacts related to climate change is increasing, with emerging enquiries and initiatives (e.g., Royal Commission into National Natural Disaster Arrangements; Commonwealth of Australia 2020), investment into efforts to develop climate-related psychosocial interventions is lacking. This paper highlights why climate-specific mental health interventions are needed and the importance of developing youth-specific programs.

## Footnotes

1 https://trialsearch.who.int and https://clinicaltrials.gov/ searched on 30th August 2024.
